# 1-Benzyl-3-phenyl­imidazolium hexa­fluoro­phosphate

**DOI:** 10.1107/S1600536809031584

**Published:** 2009-08-19

**Authors:** Ping Jiang

**Affiliations:** a School of Chemistry and Chemical Engineering, China West Normal University, Nanchong 637002, People’s Republic of China

## Abstract

in the title compound, C_16_H_15_N_2_^+^·PF_6_^−^, a precursor of *N*-heterocyclic carbene, the phenyl and benzyl rings are twisted away from the central imidazolium ring system, making dihedral angles of 70.30 (8) and 32.03 (10)°, respectively. The crystal structure is stabilized by C—H⋯F hydrogen bonds. Furthermore, P—F⋯π inter­actions involving imidazolium rings are observed [F⋯π = 2.9857 (16), P⋯π = 4.1630 (16) Å, P—F⋯π = 127.92 (6)°].

## Related literature

The first stable *N*-heterocyclic carbene was isolated by Arduengo *et al.* (1991 ▶). For the synthesis, see: Liu *et al.* (2003 ▶). For related structures, see: Wan *et al.* (2008 ▶). For related structures, see: Newman *et al.* (2007 ▶); Herrmann (2002 ▶); Yang *et al.* (2009 ▶).
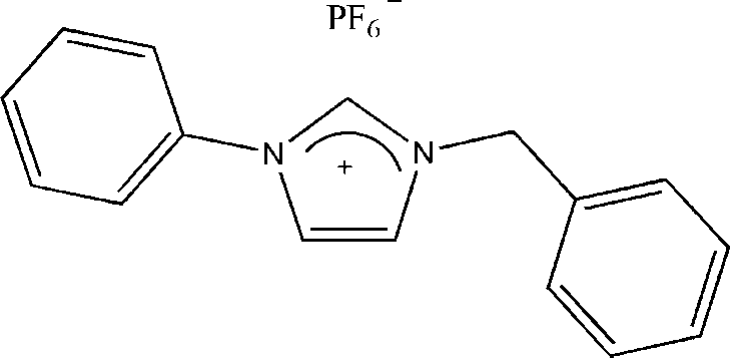


## Experimental

### 

#### Crystal data

C_16_H_15_N_2_^+^·PF_6_^−^
*M* *_r_* = 380.27
Triclinic, 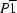

*a* = 9.221 (2) Å
*b* = 10.046 (3) Å
*c* = 10.108 (2) Å
α = 110.733 (2)°
β = 91.969 (2)°
γ = 110.315 (2)°
*V* = 807.9 (3) Å^3^
*Z* = 2
Mo *K*α radiation
μ = 0.24 mm^−1^
*T* = 93 K
0.43 × 0.40 × 0.37 mm

#### Data collection

Rigaku SPIDER diffractometer
Absorption correction: multi-scan (*ABSCOR*; Higashi, 1995 ▶) *T*_min_ = 0.905, *T*_max_ = 0.919
4739 measured reflections
2902 independent reflections
2361 reflections with *I* > 2σ(*I*)
*R*_int_ = 0.017

#### Refinement

*R*[*F* ^2^ > 2σ(*F* ^2^)] = 0.035
*wR*(*F* ^2^) = 0.084
*S* = 1.00
2902 reflections
226 parameters
H-atom parameters constrained
Δρ_max_ = 0.22 e Å^−3^
Δρ_min_ = −0.28 e Å^−3^

### 

Data collection: *RAPID-AUTO* (Rigaku/MSC, 2004 ▶); cell refinement: *RAPID-AUTO*; data reduction: *RAPID-AUTO*; program(s) used to solve structure: *SHELXS97* (Sheldrick, 2008 ▶); program(s) used to refine structure: *SHELXL97* (Sheldrick, 2008 ▶); molecular graphics: *SHELXTL* (Sheldrick, 2008 ▶); software used to prepare material for publication: *SHELXTL*.

## Supplementary Material

Crystal structure: contains datablocks global, I. DOI: 10.1107/S1600536809031584/at2854sup1.cif

Structure factors: contains datablocks I. DOI: 10.1107/S1600536809031584/at2854Isup2.hkl

Additional supplementary materials:  crystallographic information; 3D view; checkCIF report

## Figures and Tables

**Table 1:** Hydrogen-bond geometry (Å, °)

*D*—H⋯*A*	*D*—H	H⋯*A*	*D*⋯*A*	*D*—H⋯*A*
C7—H7⋯F1^i^	0.95	2.50	3.099 (2)	121
C8—H8⋯F6^i^	0.95	2.50	3.392 (2)	156
C9—H9⋯F5^ii^	0.95	2.34	3.247 (2)	159
C10—H10*A*⋯F4^ii^	0.99	2.49	3.444 (2)	161
C10—H10*B*⋯F3^iii^	0.99	2.49	3.455 (3)	164

Symmetry codes: (i) 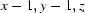; (ii) 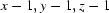; (iii) 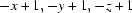.

**Fig. 1.**
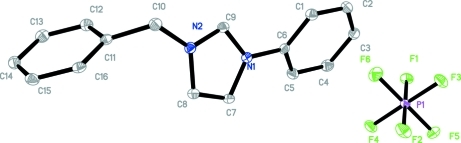
The molecular structure of the title compound, showing 30% probability displacement ellipsoids and the atomic numbering.

## supplementary crystallographic information

### Comment

Initiated by the isolation of the first stable N-heterocyclic carbene (NHC) by Arduengo *et al.* (1991), numerous stable NHC ligands has been prepared. 1,3-Disubstitutedimidazolium salts are considerable good precursor for synthesis of transition metal NHCs. In addition, the study of biological activities of imidazolium salts have been reported during these years. We report herein the synthesis and crystal structure of the title compound (I).

In (I), bond lengths and angles in title molecule (Fig. 1) are normal. The phenyl ring make dihedral angles with the benzyl ring and the imidazolium ring of 70.30 (8)° and 32.03 (10)°, respectively.

The crystal structure is stabilized by C—H···F hydrogen bonds (Table 1). Furthermore, P—F···π interactions involving imidazolium rings [F1···Cg1^iv^ = 2.9857 (16) Å, P1···Cg1^iv^ = 4.1630 (16) Å, P1—F1··· Cg1^iv^ = 127.92 (6)°, where Cg1 is a centroid of the N1/N2/C7–C9 ring; symmetry code: (iv) -x+2, -y+1, -z+1] are observed.

### Experimental

The title compound was prepared according to the reported procedure of Liu *et al.* (2003). Colourless single crystals suitable for X-ray diffraction were obtained by recrystallization from dichloromethane and pPetroleum ether.

### Refinement

H atoms were placed in calculated positions with C—H = 0.95–0.9900 Å, and refined in riding mode with *U*_iso_(H) = 1.2*U*_eq_(C).

### Crystal data

**Table:**

C_16_H_15_N_2_^+^·PF_6_^−^	*Z* = 2
*M**_r_* = 380.27	*F*(000) = 388
Triclinic, *P*1	*D*_x_ = 1.563 Mg m^−^^3^
Hall symbol: -P 1	Mo *K*α radiation, λ = 0.71073 Å
*a* = 9.221 (2) Å	Cell parameters from 2516 reflections
*b* = 10.046 (3) Å	θ = 3.1–27.5°
*c* = 10.108 (2) Å	µ = 0.24 mm^−^^1^
α = 110.733 (2)°	*T* = 93 K
β = 91.969 (2)°	Block, colourless
γ = 110.315 (2)°	0.43 × 0.40 × 0.37 mm
*V* = 807.9 (3) Å^3^	

### Data collection

**Table:**

Rigaku SPIDER diffractometer	2902 independent reflections
Radiation source: Rotating Anode	2361 reflections with *I* > 2σ(*I*)
graphite	*R*_int_ = 0.017
ω scans	θ_max_ = 25.5°, θ_min_ = 3.1°
Absorption correction: multi-scan (*ABSCOR*; Higashi, 1995)	*h* = −11→7
*T*_min_ = 0.905, *T*_max_ = 0.919	*k* = −11→12
4739 measured reflections	*l* = −12→11

### Refinement

**Table:**

Refinement on *F*^2^	Primary atom site location: structure-invariant direct methods
Least-squares matrix: full	Secondary atom site location: difference Fourier map
*R*[*F*^2^ > 2σ(*F*^2^)] = 0.035	Hydrogen site location: inferred from neighbouring sites
*wR*(*F*^2^) = 0.084	H-atom parameters constrained
*S* = 1.00	*w* = 1/[σ^2^(*F*_o_^2^) + (0.043*P*)^2^ + 0.066*P*] where *P* = (*F*_o_^2^ + 2*F*_c_^2^)/3
2902 reflections	(Δ/σ)_max_ < 0.001
226 parameters	Δρ_max_ = 0.22 e Å^−^^3^
0 restraints	Δρ_min_ = −0.28 e Å^−^^3^

### Special details

**Table:**

Geometry. All e.s.d.'s (except the e.s.d. in the dihedral angle between two l.s. planes) are estimated using the full covariance matrix. The cell e.s.d.'s are taken into account individually in the estimation of e.s.d.'s in distances, angles and torsion angles; correlations between e.s.d.'s in cell parameters are only used when they are defined by crystal symmetry. An approximate (isotropic) treatment of cell e.s.d.'s is used for estimating e.s.d.'s involving l.s. planes.
Refinement. Refinement of *F*^2^ against ALL reflections. The weighted *R*-factor *wR* and goodness of fit *S* are based on *F*^2^, conventional *R*-factors *R* are based on *F*, with *F* set to zero for negative *F*^2^. The threshold expression of *F*^2^ > σ(*F*^2^) is used only for calculating *R*-factors(gt) *etc*. and is not relevant to the choice of reflections for refinement. *R*-factors based on *F*^2^ are statistically about twice as large as those based on *F*, and *R*- factors based on ALL data will be even larger.

### Fractional atomic coordinates and isotropic or equivalent isotropic displacement parameters (Å^2^)

**Table:**

	*x*	*y*	*z*	*U*_iso_*/*U*_eq_	
P1	1.23197 (5)	1.00569 (5)	0.79232 (5)	0.02127 (14)	
F1	1.38948 (11)	1.06413 (12)	0.73171 (11)	0.0297 (3)	
F2	1.07593 (13)	0.94747 (15)	0.85307 (13)	0.0444 (3)	
F3	1.16399 (13)	1.10464 (14)	0.73467 (12)	0.0380 (3)	
F4	1.30246 (13)	0.90834 (14)	0.85235 (12)	0.0381 (3)	
F5	1.30920 (14)	1.15038 (13)	0.94289 (11)	0.0427 (3)	
F6	1.15883 (14)	0.86186 (13)	0.64242 (12)	0.0433 (3)	
N1	0.47043 (15)	0.20046 (16)	0.35708 (14)	0.0188 (3)	
N2	0.28468 (15)	−0.02584 (16)	0.25378 (14)	0.0192 (3)	
C1	0.5504 (2)	0.4242 (2)	0.29382 (18)	0.0235 (4)	
H1	0.4593	0.3740	0.2211	0.028*	
C2	0.6550 (2)	0.5713 (2)	0.31694 (19)	0.0255 (4)	
H2	0.6367	0.6216	0.2581	0.031*	
C3	0.7858 (2)	0.6454 (2)	0.42503 (18)	0.0241 (4)	
H3	0.8553	0.7475	0.4422	0.029*	
C4	0.8153 (2)	0.5709 (2)	0.50786 (19)	0.0270 (4)	
H4	0.9061	0.6213	0.5810	0.032*	
C5	0.7131 (2)	0.4229 (2)	0.48495 (19)	0.0243 (4)	
H5	0.7337	0.3714	0.5416	0.029*	
C6	0.58132 (19)	0.3514 (2)	0.37897 (18)	0.0191 (4)	
C7	0.4319 (2)	0.1417 (2)	0.46130 (18)	0.0218 (4)	
H7	0.4787	0.1912	0.5602	0.026*	
C8	0.3160 (2)	0.0014 (2)	0.39716 (18)	0.0223 (4)	
H8	0.2652	−0.0662	0.4423	0.027*	
C9	0.37939 (19)	0.0950 (2)	0.23205 (18)	0.0194 (4)	
H9	0.3822	0.1051	0.1421	0.023*	
C10	0.1627 (2)	−0.1631 (2)	0.14214 (19)	0.0233 (4)	
H10A	0.1772	−0.1595	0.0467	0.028*	
H10B	0.0580	−0.1612	0.1581	0.028*	
C11	0.16757 (19)	−0.3114 (2)	0.14213 (17)	0.0197 (4)	
C12	0.28553 (19)	−0.3616 (2)	0.08983 (18)	0.0224 (4)	
H12	0.3691	−0.2983	0.0598	0.027*	
C13	0.2811 (2)	−0.5030 (2)	0.08160 (18)	0.0254 (4)	
H13	0.3614	−0.5368	0.0453	0.030*	
C14	0.1600 (2)	−0.5965 (2)	0.12598 (18)	0.0255 (4)	
H14	0.1569	−0.6941	0.1195	0.031*	
C15	0.0439 (2)	−0.5460 (2)	0.17967 (18)	0.0248 (4)	
H15	−0.0385	−0.6087	0.2113	0.030*	
C16	0.04776 (19)	−0.4049 (2)	0.18733 (17)	0.0219 (4)	
H16	−0.0325	−0.3713	0.2240	0.026*	

### Atomic displacement parameters (Å^2^)

**Table:**

	*U*^11^	*U*^22^	*U*^33^	*U*^12^	*U*^13^	*U*^23^
P1	0.0232 (3)	0.0215 (3)	0.0217 (3)	0.0089 (2)	0.0073 (2)	0.0108 (2)
F1	0.0233 (5)	0.0358 (7)	0.0297 (6)	0.0075 (5)	0.0084 (5)	0.0159 (5)
F2	0.0366 (7)	0.0571 (9)	0.0629 (8)	0.0237 (6)	0.0301 (6)	0.0422 (7)
F3	0.0390 (7)	0.0481 (8)	0.0470 (7)	0.0250 (6)	0.0148 (6)	0.0323 (7)
F4	0.0498 (7)	0.0469 (8)	0.0437 (7)	0.0330 (6)	0.0230 (6)	0.0315 (6)
F5	0.0644 (8)	0.0339 (7)	0.0229 (6)	0.0196 (6)	0.0073 (6)	0.0029 (6)
F6	0.0445 (7)	0.0274 (7)	0.0348 (7)	−0.0020 (6)	−0.0008 (5)	0.0025 (6)
N1	0.0206 (7)	0.0181 (8)	0.0174 (8)	0.0075 (6)	0.0029 (6)	0.0066 (7)
N2	0.0197 (7)	0.0187 (8)	0.0184 (8)	0.0076 (6)	0.0021 (6)	0.0063 (7)
C1	0.0236 (9)	0.0251 (10)	0.0214 (10)	0.0088 (8)	0.0026 (8)	0.0094 (8)
C2	0.0305 (10)	0.0247 (10)	0.0259 (10)	0.0122 (8)	0.0077 (8)	0.0134 (9)
C3	0.0251 (9)	0.0209 (10)	0.0239 (10)	0.0065 (8)	0.0083 (8)	0.0081 (8)
C4	0.0244 (9)	0.0275 (11)	0.0228 (10)	0.0061 (8)	−0.0002 (8)	0.0070 (9)
C5	0.0264 (9)	0.0238 (10)	0.0227 (10)	0.0086 (8)	0.0015 (8)	0.0103 (9)
C6	0.0211 (9)	0.0178 (9)	0.0177 (9)	0.0081 (7)	0.0058 (7)	0.0053 (8)
C7	0.0308 (10)	0.0232 (10)	0.0148 (9)	0.0128 (8)	0.0059 (7)	0.0086 (8)
C8	0.0292 (10)	0.0234 (10)	0.0174 (9)	0.0121 (8)	0.0070 (8)	0.0093 (8)
C9	0.0203 (8)	0.0216 (10)	0.0164 (9)	0.0088 (7)	0.0023 (7)	0.0068 (8)
C10	0.0210 (9)	0.0238 (10)	0.0216 (10)	0.0059 (8)	−0.0007 (7)	0.0078 (8)
C11	0.0202 (8)	0.0204 (10)	0.0146 (9)	0.0059 (7)	−0.0017 (7)	0.0049 (8)
C12	0.0187 (9)	0.0248 (10)	0.0218 (10)	0.0060 (8)	0.0038 (7)	0.0092 (8)
C13	0.0233 (9)	0.0292 (11)	0.0229 (10)	0.0120 (8)	−0.0007 (8)	0.0079 (9)
C14	0.0293 (10)	0.0209 (10)	0.0218 (10)	0.0065 (8)	−0.0052 (8)	0.0073 (8)
C15	0.0225 (9)	0.0265 (11)	0.0205 (10)	0.0019 (8)	−0.0006 (8)	0.0116 (9)
C16	0.0200 (9)	0.0264 (10)	0.0147 (9)	0.0063 (8)	0.0007 (7)	0.0057 (8)

### Geometric parameters (Å, °)

**Table:**

P1—F2	1.5875 (11)	C5—C6	1.378 (2)
P1—F3	1.5933 (11)	C5—H5	0.9500
P1—F6	1.5942 (12)	C7—C8	1.345 (2)
P1—F1	1.5978 (11)	C7—H7	0.9500
P1—F5	1.6052 (12)	C8—H8	0.9500
P1—F4	1.6066 (11)	C9—H9	0.9500
N1—C9	1.336 (2)	C10—C11	1.505 (2)
N1—C7	1.380 (2)	C10—H10A	0.9900
N1—C6	1.437 (2)	C10—H10B	0.9900
N2—C9	1.323 (2)	C11—C16	1.389 (2)
N2—C8	1.376 (2)	C11—C12	1.396 (2)
N2—C10	1.474 (2)	C12—C13	1.379 (2)
C1—C2	1.385 (2)	C12—H12	0.9500
C1—C6	1.389 (2)	C13—C14	1.391 (2)
C1—H1	0.9500	C13—H13	0.9500
C2—C3	1.383 (2)	C14—C15	1.386 (2)
C2—H2	0.9500	C14—H14	0.9500
C3—C4	1.380 (2)	C15—C16	1.380 (2)
C3—H3	0.9500	C15—H15	0.9500
C4—C5	1.386 (2)	C16—H16	0.9500
C4—H4	0.9500		
			
F2—P1—F3	90.46 (6)	C5—C6—C1	121.20 (17)
F2—P1—F6	90.77 (7)	C5—C6—N1	120.21 (15)
F3—P1—F6	90.53 (7)	C1—C6—N1	118.58 (15)
F2—P1—F1	179.73 (6)	C8—C7—N1	107.46 (15)
F3—P1—F1	89.79 (6)	C8—C7—H7	126.3
F6—P1—F1	89.34 (6)	N1—C7—H7	126.3
F2—P1—F5	90.23 (7)	C7—C8—N2	107.13 (15)
F3—P1—F5	90.14 (7)	C7—C8—H8	126.4
F6—P1—F5	178.80 (7)	N2—C8—H8	126.4
F1—P1—F5	89.66 (6)	N2—C9—N1	108.96 (14)
F2—P1—F4	89.83 (6)	N2—C9—H9	125.5
F3—P1—F4	179.11 (7)	N1—C9—H9	125.5
F6—P1—F4	90.31 (7)	N2—C10—C11	112.54 (14)
F1—P1—F4	89.92 (6)	N2—C10—H10A	109.1
F5—P1—F4	89.02 (7)	C11—C10—H10A	109.1
C9—N1—C7	107.81 (14)	N2—C10—H10B	109.1
C9—N1—C6	125.48 (14)	C11—C10—H10B	109.1
C7—N1—C6	126.60 (15)	H10A—C10—H10B	107.8
C9—N2—C8	108.63 (14)	C16—C11—C12	118.95 (16)
C9—N2—C10	124.79 (14)	C16—C11—C10	119.61 (15)
C8—N2—C10	126.53 (14)	C12—C11—C10	121.34 (15)
C2—C1—C6	118.79 (17)	C13—C12—C11	120.18 (16)
C2—C1—H1	120.6	C13—C12—H12	119.9
C6—C1—H1	120.6	C11—C12—H12	119.9
C3—C2—C1	120.50 (17)	C12—C13—C14	120.50 (17)
C3—C2—H2	119.8	C12—C13—H13	119.8
C1—C2—H2	119.8	C14—C13—H13	119.8
C4—C3—C2	119.89 (17)	C15—C14—C13	119.44 (17)
C4—C3—H3	120.1	C15—C14—H14	120.3
C2—C3—H3	120.1	C13—C14—H14	120.3
C3—C4—C5	120.38 (17)	C16—C15—C14	120.11 (16)
C3—C4—H4	119.8	C16—C15—H15	119.9
C5—C4—H4	119.8	C14—C15—H15	119.9
C6—C5—C4	119.21 (17)	C15—C16—C11	120.81 (16)
C6—C5—H5	120.4	C15—C16—H16	119.6
C4—C5—H5	120.4	C11—C16—H16	119.6
			
C6—C1—C2—C3	−1.4 (3)	C8—N2—C9—N1	−0.49 (18)
C1—C2—C3—C4	1.9 (3)	C10—N2—C9—N1	177.44 (14)
C2—C3—C4—C5	−1.0 (3)	C7—N1—C9—N2	0.77 (18)
C3—C4—C5—C6	−0.3 (3)	C6—N1—C9—N2	−175.77 (14)
C4—C5—C6—C1	0.8 (3)	C9—N2—C10—C11	132.27 (16)
C4—C5—C6—N1	−177.50 (15)	C8—N2—C10—C11	−50.2 (2)
C2—C1—C6—C5	0.0 (3)	N2—C10—C11—C16	109.96 (17)
C2—C1—C6—N1	178.40 (15)	N2—C10—C11—C12	−73.7 (2)
C9—N1—C6—C5	−151.02 (16)	C16—C11—C12—C13	0.9 (2)
C7—N1—C6—C5	33.1 (2)	C10—C11—C12—C13	−175.41 (16)
C9—N1—C6—C1	30.6 (2)	C11—C12—C13—C14	−0.4 (3)
C7—N1—C6—C1	−145.30 (16)	C12—C13—C14—C15	−0.4 (3)
C9—N1—C7—C8	−0.76 (18)	C13—C14—C15—C16	0.7 (2)
C6—N1—C7—C8	175.73 (15)	C14—C15—C16—C11	−0.2 (2)
N1—C7—C8—N2	0.46 (18)	C12—C11—C16—C15	−0.6 (2)
C9—N2—C8—C7	0.01 (18)	C10—C11—C16—C15	175.78 (15)
C10—N2—C8—C7	−177.88 (15)		

### Hydrogen-bond geometry (Å, °)

**Table:**

*D*—H···*A*	*D*—H	H···*A*	*D*···*A*	*D*—H···*A*
C7—H7···F1^i^	0.95	2.50	3.099 (2)	121
C8—H8···F6^i^	0.95	2.50	3.392 (2)	156
C9—H9···F5^ii^	0.95	2.34	3.247 (2)	159
C10—H10A···F4^ii^	0.99	2.49	3.444 (2)	161
C10—H10B···F3^iii^	0.99	2.49	3.455 (3)	164

Symmetry codes: (i) *x*−1, *y*−1, *z*; (ii) *x*−1, *y*−1, *z*−1; (iii) −*x*+1, −*y*+1, −*z*+1.
